# Effects of Glycemic Gap on Post-Stroke Cognitive Impairment in Acute Ischemic Stroke Patients

**DOI:** 10.3390/brainsci11050612

**Published:** 2021-05-11

**Authors:** Minwoo Lee, Jae-Sung Lim, Yerim Kim, Ju Hun Lee, Chul-Ho Kim, Sang-Hwa Lee, Min Uk Jang, Mi Sun Oh, Byung-Chul Lee, Kyung-Ho Yu

**Affiliations:** 1 Department of Neurology, Hallym Neurological Institute, Hallym University College of Medicine, Hallym University Sacred Heart Hospital, Anyang 14068, Korea; minwoo.lee.md@gmail.com (M.L.); iyyar@hallym.or.kr (M.S.O.); ssbrain@hallym.or.kr (B.-C.L.); 2 Asan Medical Center, Department of Neurology, Seoul 05505, Korea; 3 Department of Neurology, Hallym Neurological Institute, Hallym University College of Medicine, Kangdong Sacred Heart Hospital, Seoul 05355, Korea; brainyrk@gmail.com (Y.K.); leeforte@paran.com (J.H.L.); 4 Department of Neurology, Hallym Neurological Institute, Hallym University College of Medicine, Chuncheon Sacred Heart Hospital, Chunchon 24253, Korea; gumdol52@naver.com (C.-H.K.); bleulsh@naver.com (S.-H.L.); 5 Department of Neurology, Hallym Neurological Institute, Hallym University College of Medicine, Dongtan Sacred Heart Hospital, Hwaseong 18450, Korea; mujang@gmail.com

**Keywords:** glycemic gap, hemoglobin A1c, stroke, cerebral infarction, cognitive impairment

## Abstract

Background: Post-stroke hyperglycemia is a frequent finding in acute ischemic stroke patients and is associated with poor functional and cognitive outcomes. However, it is unclear as to whether the glycemic gap between the admission glucose and HbA1c-derived estimated average glucose (eAG) is associated with post-stroke cognitive impairment (PSCI). Methods: We enrolled acute ischemic stroke patients whose cognitive functions were evaluated three months after a stroke using the Korean version of the vascular cognitive impairment harmonization standards neuropsychological protocol (K-VCIHS-NP). The development of PSCI was defined as having z-scores of less than −2 standard deviations in at least one cognitive domain. The participants were categorized into three groups according to the glycemic gap status: non-elevated (initial glucose − eAG ≤ 0 mg/dL), mildly elevated (0 mg/dL < initial glucose − eAG < 50 mg/dL), and severely elevated (50 mg/dL ≤ initial glucose − eAG). Results: A total of 301 patients were enrolled. The mean age was 63.1 years, and the median National Institute of Health Stroke Scale (NIHSS) score was two (IQR: 1–4). In total, 65 patients (21.6%) developed PSCI. In multiple logistic regression analyses, the severely elevated glycemic gap was a significant predictor for PSCI after adjusting for age, sex, education level, initial stroke severity, Trial of Org 10172 in Acute Stroke Treatment (TOAST) classification, and left hemispheric lesion (aOR: 3.65, *p*-value = 0.001). Patients in the severely elevated glycemic gap group showed significantly worse performance in the frontal and memory domains. Conclusions: In conclusion, our study demonstrated that an elevated glycemic gap was significantly associated with PSCI three months after a stroke, with preferential involvement of frontal and memory domain dysfunctions.

## 1. Introduction

Post-stroke cognitive impairment (PSCI) is one of the major disabilities encountered by post-stroke survivors. As a potential risk factor for PSCI, multiple glucose parameters, such as hemoglobin A1c, hyperglycemia, glycemic variability, and glucose dynamics, have received significant attention for their implication in both diabetic and non-diabetic patients [1,2,3]. Although the association between admission hyperglycemia and PSCI still remains controversial, post-stroke hyperglycemia has been shown to be associated with PSCI in non-diabetic and fairly well-controlled diabetic patients [2]. Though speculative, hyperglycemia may affect cognition after stroke by the aggravation of the secondary neuronal injury cascades, including increased production of reactive oxygen species, inflammation, mitochondrial dysfunction, and microglial activation [4].

However, hyperglycemia should be considered along with the pre-existing glycemic control status to reflect acute physiological stress. For this purpose, previous studies have utilized the glycemic gap as a surrogate marker for stress-induced hyperglycemia (SIH) by subtracting the HbA1c-derived estimated average glucose (eAG) from the initial serum glucose [5,6,7,8]. A recent study revealed that an elevated glycemic gap upon admission is a significant predictor for poor functional outcomes three months after a stroke [9]. The study also showed that the association between them remained significant only in non-diabetic patients and diabetic patients with good glycemic control status. They suggested that an elevated glycemic gap during the acute stage better reflects stress-induced hyperglycemia (SIH) and is a better prognostic factor than hyperglycemia itself without considering the glycemic control status.

As such, we hypothesized that the effects of an elevated glycemic gap during the acute stage of a stroke are likely to serve as an important predictor of, as well as a potential therapeutic target for, cognitive outcomes after a stroke. While the glycemic gap has been studied as a prognostic factor for functional outcomes after a stroke, the association between the glycemic gap and PSCI has not been established. Thus, we investigated the correlation between the elevated glycemic gap and the development of post-stroke cognitive impairment after a stroke, with a focus on its differential effects on domain-specific cognitive outcomes.

## 2. Materials and Methods

### 2.1. Study Design and Population

This observational retrospective study, based on a prospective stroke registry from a tertiary university hospital, assessed the association between the glycemic gap on admission and PSCI in ischemic stroke patients. The study was approved by the institutional review board of Hallym University Sacred Heart Hospital and conformed to the Declaration of Helsinki. The requirement for patient consent was waived because of the retrospective nature of this study, as well as the minimal risk that it posed to participants.

We enrolled participants with the following inclusion criteria: (1) a diagnosis of acute ischemic stroke with a relevant lesion confirmed on diffusion-weighted imaging; (2) admission within 7 days of symptom onset; (3) available data on hemoglobin A1c and the initial serum glucose; and (4) available data on a neuropsychological test battery 3 months after stroke onset. Participants with premorbid cognitive impairment (IQCODE > 3.6) or functional disability (modified Rankin scale score of >2) were excluded. A total of 301 participants were analyzed. All patients received standard acute stroke management, including optimal glycemic control, during hospitalization.

### 2.2. Clinical Variables

Baseline demographic factors, including age, sex, education level, and vascular risk factors (hypertension, diabetes mellitus, dyslipidemia, atrial fibrillation, and smoking status) were collected. Clinical characteristics, including the initial stroke severity assessed with a National Institutes of Health Stroke Scale (NIHSS) score and stroke subtype, according to the Trial of Org 10172 in Acute Stroke Treatment (TOAST) classification [10], were also assessed.

All participants underwent 3T brain magnetic resonance imaging, and acute cerebral infarction was confirmed with diffusion-weighted images. The number and location of ischemic lesions was assessed, quantified and then subdivided as follows: (1) single vs. multiple, (2) cortical vs. subcortical, and (3) the presence of left hemispheric lesions [11].

The initial serum glucose level was obtained upon admission to the emergency department, and the hemoglobin A1c level was obtained after an overnight fast within 24 h after admission. The equation for the eAG was calculated using a validated formula to convert HbA1C levels to the eAG for the preceding 3 months [12]

eAG (mg/dL) = 28.7 × HbA1c (mg/dL) − 46.7

The glycemic gap represents the differences between the initial glucose level at admission and the eAG. The initial glucose level minus the eAG was calculated, and the participants were divided into three groups: non-elevated (initial glucose − eAG ≤ 0 mg/dL), mildly elevated (0 mg/dL < initial glucose − eAG < 50 mg/dL) and severely elevated (50 mg/dL ≤ initial glucose − eAG). The glycemic control status was divided into good (HbA1c < 6.5%) and poor (HbA1c ≥ 6.5%) groups.

### 2.3. Neuropsychological Outcome Variables

We performed a 60-min neuropsychological test battery using the Korean version of the vascular cognitive impairment harmonization standards neuropsychological protocol (K-VCIHS-NP) 3 months after patients experienced a stroke [13]. The K-VCIHS-NP assesses four major cognitive functions: the memory, frontal, visuospatial, and language domains [13,14]. The frontal function was evaluated with the Korean version of the controlled oral word association test, semantic fluency (animal naming) [15], digit symbol coding [16], and the Korean version of the trail-making test for elderly patients [17]. The tests for other cognitive functions were the Seoul verbal learning test for memory function, the Korean version of the Boston naming test (short form A for language function) [18], and the Rey complex figure test copy score for visuospatial function [19]. We also performed the Korean version of the mini-mental state examination (K-MMSE) to assess global cognitive function [20]. All cognitive tests included in the K-VCIHS-NP were validated and standardized for use in the Korean population [13], and the scores of each cognitive test were adjusted for age, sex, and education level and then transformed into z-scores (i.e., normalized scores) for analysis. All four major domain-specific z-scores were also assessed. The primary outcome was the development of PSCI at 3 months, which was defined as having z-scores of less than −2 standard deviations in at least one cognitive domain [21].

### 2.4. Statistical Analysis

Continuous variables were expressed as the mean ± standard deviation or median with an interquartile range as appropriate, and categorical variables were expressed as frequencies. The distribution of demographics, clinical characteristics, and stroke subtypes between the no cognitive impairment group and the PSCI group were analyzed using the Student’s *t*-test or the Mann–Whitney U test for continuous variables and the chi-squared test or Fisher’s exact test for categorical variables as appropriate. Clinical characteristics among three glycemic gap groups were also compared using the analysis of variance, the Kruskal–Wallis test, or the chi-squared test as appropriate.

Associations between the glycemic gap and 3-month PSCI were investigated using univariable and multivariable logistic regression analyses. Multivariable models were adjusted for variables with *p*-values of <0.1 in univariate analysis and the prespecified variables including age, sex, education, initial stroke severity (NIHSS), and stroke subtype (TOAST). A secondary analysis was also performed to assess the differential effects of the glycemic gap both in good and poor glycemic control status [9]. In both analyses, the non-elevated glycemic gap group (initial glucose − eAG ≤ 0 mg/dL) was used as a reference. An association was indicated with the odds ratio and adjusted odds ratio (aOR) with a 95% confidence interval (CI). Analysis of covariance was conducted to compare the z-scores of each domain and global cognitive functions among three glycemic gap groups, adjusting for age, sex, education level, and initial stroke severity (NIHSS). All statistical analyses were conducted using R (version 4.0.3; R Foundation for Statistical Computing), and two-tailed *p*-values of <0.05 were considered statistically significant.

## 3. Results

A total of 301 subjects were included in our study. The mean age was 63.1 years (standard deviation: 12.1 years), and the average interval between stroke onset and performance of a neuropsychological test using the K-VCIHS-NP was 103.1 days (standard deviation: 14.4 days). The median initial NIHSS score was 2 (interquartile range: 1–4). In total, 65 patients (21.6%) developed PSCI 3 months after having an ischemic stroke. The baseline characteristics between no cognitive impairment (NCI) group and PSCI group are presented in the Table 1.

In multiple logistic regression analyses, the severely elevated glycemic gap was a significant predictor for PSCI, after adjusting for age, sex, education level, initial stroke severity, TOAST classification, and presence of a left hemispheric lesion (aOR: 3.65; 95% CI, 1.65–8.06; Table 2). The initial NIHSS score was also significantly associated with PSCI (aOR: 1.09; 95% CI, 1.02–1.17). We also conducted subgroup analysis after categorization of participants by glycemic control status (good: HbA1c < 6.5%; poor: HbA1c ≥ 6.5%) [9] after adjusting for the covariates used in the main analysis. The association between the severely elevated glycemic gap and PSCI remained significant in the good glycemic control status group (aOR: 4.58; 95% CI, 1.31–16.02; Table 3), whereas the association was lost in the poor glycemic control status group (aOR: 2.58; 95% CI, 0.81–8.16).

We also compared the global cognitive functions and all four major cognitive domains among the three glycemic groups using analysis of covariance. Three months after a stroke, the z-scores of the frontal and memory domains were significantly different between the groups after adjusting for age, sex, education level, and initial NIHSS scores (*p* = 0.002 and *p* = 0.023, respectively). Multiple comparison analysis showed that the severely elevated glycemic gap group had lower frontal and memory z-scores compared with both the non-elevated and mildly elevated groups. (Table 4).

## 4. Discussion

In the current study, we demonstrated that an elevated glycemic gap on admission in an acute stroke setting was independently associated with an increased risk of developing PSCI. Both the frontal and memory domains were specifically affected among patients with an elevated glycemic gap. In a subgroup analysis, considering the glycemic control status with HbA1c as a parameter, the association between an elevated glycemic gap and PSCI remained significant only in the good glycemic control status group (HbA1c < 6.5%).

Acute hyperglycemia after a stroke may be due to preexisting abnormalities in glucose metabolism, including prediabetes and diabetes mellitus, or it may reflect an acute stress response [22]. Several potential pathophysiology examples have been suggested for the observed association between the glycemic gap and poor cognitive function after a stroke. First, hyperglycemia may induce lactate accumulation and intracellular acidosis in the ischemic brain [23]. The resultant acidosis enhances lipid peroxidation, production of reactive oxygen species, and mitochondrial dysfunction, which aggravates secondary injury to the ischemic brain [24]. Second, hyperglycemia in patients without a previous diagnosis of diabetes are likely to have insulin resistance or impaired glucose tolerance [25], which are also known to be associated with aggravated secondary neuronal injury after ischemic stroke via the reduced peripheral uptake of glucose and endothelial dysfunction [26]. Third, SIH may be a marker of the severity of ischemic insult in patients with strokes. Patients with massive ischemia may develop reactive hyperglycemia due to the release of acute stress hormones such as cortisol. However, SIH is not only an epiphenomenon of severe stroke, but also an effector of further neuronal injury, as animal studies have shown that controlling hyperglycemia with insulin reduced the size of the ischemic lesion and improved functional outcomes after a stroke [27].

Most previous studies which investigated hyperglycemia and cognitive impairment after a stroke focused on the initial glucose level or fasting glucose level without considering the estimated glucose level at the time of a stroke’s onset [2,28]. Our previous study revealed that hyperglycemia was associated with a 3-month cognitive outcome in non-diabetic or diabetic patients with HbA1c levels of less than 8.0% [2]. On the contrary, a study with the European stroke population revealed that hyperglycemia was not associated with impaired cognition after 6–10 months [28]. Discrepancies in these findings may be due to differences in study populations, design, neuropsychological tests, but most importantly, the definition of hyperglycemia and consideration of glycemic control status. Thus, our findings may add to the hypothesis that SIH, represented by the glycemic gap, may better explain short-term cognitive outcomes after a stroke than hyperglycemia itself.

Our subgroup analysis revealed that the association between the glycemic gap and post-stroke cognitive impairment was lost in a poorly controlled glycemic status, with HbA1c > 6.5%. This was in line with our previous study in that hyperglycemia is associated with cognitive outcomes only in non-diabetic or well-controlled diabetic patients. There are several potential explanations for this finding. First, patients with uncontrolled diabetes may already be preconditioned to hyperglycemia and thus might have different threshold values for poor cognitive outcomes [29]. Second, diabetic patients are more likely to be treated for hyperglycemia properly in the acute stage of a stroke during admission and after discharge. Anti-diabetic medications may reduce lactic acidosis and other metabolic derangements in the brain, leading to the alleviation of short-term cognitive dysfunction. However, this finding should be interpreted with caution, as poorly controlled diabetes can adversely affect long-term cognitive functions through multiple vascular complications.

Additional analysis on domain-specific cognitive outcomes revealed that an elevated glycemic gap was associated with lower z-scores of the frontal and memory domains. The findings were in line with previous studies with type 2 diabetes mellitus and cognitive outcome after a stroke, in that T2DM was associated with all major cognitive domains with the predilection of frontal domains [3]. Our previous study also revealed that glycemic variability was associated with frontal and memory dysfunctions after a stroke [2]. Disorders of glucose metabolism, including T2DM, hyperglycemia, glycemic gap, and glycemic variabilities, may share common pathophysiological cascades, resulting in cortical atrophies and neural network dysfunction within lesions associated with memory and executive functions.

The presence of chronic brain pathologies, including cerebral small vessel disease or brain atrophy, may also have mediated the association between the glycemic gap status and PSCI. The white matter hyperintensities are important effectors of post-stroke functional recovery [30], and brain atrophy is also closely associated with pathomechanisms of the development of PSCI [31]. These chronic brain pathologies are also associated with insulin resistance and diabetes. The reduced insulin activity hinders the maintenance of myelin survival by oligodendrocytes and enhances the production of reactive oxygen species or lipid peroxide and the pro-inflammatory cytokines [32]. Insulin resistance also promotes the damage of the blood–brain barrier [33]. The combined effects of insulin resistance cause damage to myelin and the development of white matter hyperintensities. With this pathological background, recent studies have shown that intranasal insulin administration inhibits the progression of white matter lesions [34]. The direct association between the glycemic gap and brain atrophy has not been studied yet, but the association between T2DM and hippocampal atrophy regardless of vascular pathology has been observed [35]. Future studies considering the degree of brain atrophy or cerebral small vessel disease as mediators between the association between the glycemic gap and PSCI are warranted.

The strength of this study is that it is the first to investigate the effect of the glycemic gap on post-stroke cognitive impairment. Moreover, we analyzed all four major cognitive domains based on a neuropsychological test battery developed for stroke survivors and used standardized z-scores for proper comparison. However, this study does have several limitations. First, the association between the glycemic gap and PSCI could not be generalized to all stroke patients, since those with severe strokes or aphasia who could not perform neuropsychological battery were excluded. Second, several imaging variables, including the degree of cerebral small vessel diseases, brain atrophy, specific lesion location, and stroke volume, were not evaluated due to the retrospective nature of this study, and thus we were not able to correlate domain-specific cognitive dysfunction with specific lesion locations. However, we used imaging variables that were previously determined to be independent predictors of PSCI: cortical, left, and multiple lesions. Third, our study could not establish a causal relationship between the glycemic gap and PSCI due to the cross-sectional observation design. Thus, the interpretation of our findings should be limited to the consideration of the glycemic gap as a potential therapeutic target in a stroke’s acute phase. Despite these limitations, our study has its clinical significance in that this is the first study to investigate the association between the glycemic gap for the development of PSCI three months after a stroke.

## 5. Conclusions

In conclusion, our study demonstrated that an elevated glycemic gap is significantly associated with PSCI three months after a stroke, with preferential involvement of frontal and memory domain dysfunctions. As such, hyperglycemia should be considered along with the glycemic control status at the time of stroke onset for prediction of the cognitive outcome after an ischemic stroke. Further prospective longitudinal studies with larger sample size and lesion–symptom mapping should be performed to establish the causality between them.

## Figures and Tables

**Table 1:** Baseline characteristics according to the post-stroke cognitive status.

	NCI (*n* = 236)	PSCI (*n* = 65)	*p*-Value
Glycemic Gap (mg/dL)	7.5 ± 55.1	23.2 ± 59.2	0.045
Glycemic Gap Groups			0.001
Non-Elevated	129 (54.7%)	24 (36.9%)	
Mildly Elevated	80 (33.9%)	22 (33.8%)	
Severely Elevated	27 (11.4%)	19 (29.2%)	
Age (years)	62.2 ± 12.2	66.4 ± 11.2	0.013
Male Sex (%)	160 (67.8%)	35 (53.8%)	0.053
Education	9.6 ± 5.4	8.7 ± 4.6	0.251
Hypertension	135 (57.2%)	42 (64.6%)	0.351
Diabetes	63 (26.7%)	23 (35.4%)	0.223
Hyperlipidemia	54 (22.9%)	8 (12.3%)	0.090
Smoking	105 (44.5%)	32 (49.2%)	0.590
Atrial Fibrillation	6 (2.5%)	4 (6.2%)	0.295
Coronary Artery Disease	12 (5.1%)	5 (7.7%)	0.615
Initial Stroke Severity (NIHSS)	3.0 ± 3.6	5.3 ± 5.2	0.001
TOAST			0.018
-SVO	117 (50.0%)	18 (28.1%)	
-LAA	87 (37.2%)	33 (51.6%)	
-CE	10 (4.3%)	7 (10.9%)	
-OD	4 (1.7%)	1 (1.6%)	
-UD	16 (6.8%)	5 (7.8%)	
Lesion Location			
-Left Hemispheric Lesion	60 (25.4%)	26 (40.0%)	0.032
-Subcortical Lesion	44 (18.6%)	10 (15.4%)	0.672
-Multiple Lesion	12 (5.1%)	5 (7.7%)	0.615
Initial Serum Glucose (mg/dL)	143.5 ± 64.3	167.9 ± 70.5	0.008
HbA1c (%)	6.4 ± 1.5	6.7 ± 1.6	0.163

Abbreviations. NCI: no cognitive impairment; PSCI: post-stroke cognitive impairment; NIHSS: National Institute of Health Stroke Scale; TOAST: Trial of. Org 10172 in Acute Stroke Treatment; LAA: large artery atherosclerosis; SVO: small vessel occlusion; CE: cardioembolism; OD: other determined; and UD: undetermined.

**Table 2:** Multivariable analysis for possible predictors of PSCI.

	Crude OR (95% CI)	*p*-Value	Adjusted OR (95% CI)	*p*-Value
Glycemic gap				
Non-Elevated	Reference		Reference	
Mildly Elevated	1.48 (0.78–2.81)	0.233	1.41 (0.71–2.80)	0.326
Severely Elevated	3.78 (1.82–7.86)	<0.001	3.65 (1.65–8.06)	0.001
Age (per 10-year increase)	1.34 (1.10–1.63)	0.014	1.34 (1.00–1.79)	0.062
Initial NIHSS	1.12 (1.06–1.19)	<0.001	1.09 (1.02–1.17)	0.018
TOAST				
-SVO	Reference		Reference	
-LAA	2.47 (1.30–4.67)	0.006	2.01 (1.00–4.05)	0.052
-CE	4.55 (1.54–13.48)	0.006	3.07 (0.19–10.35)	0.071
-OD	1.62 (0.17–15.37)	0.671	1.51 (0.13–17.59)	0.741
-UD	2.03 (0.66–6.23)	0.215	1.81 (0.54–6.13)	0.338
Left Hemispheric	1.96 (1.10–3.48)	0.023	1.84 (0.97–3.49)	0.064

Abbreviations. OR: odds ratio; CI: confidence interval. * Adjusted for glycemic gap, age, sex, education, initial NIHSS scores, stroke subtype (TOAST), and left hemispheric lesion.

**Table 3:** Multivariable logistic regression for PSCI after categorization by glycemic control status.

	Adjusted OR (95% CI)	*p*-Value
**HbA1c < 6.5%**		
Non-elevated glycemic gap	Reference	
Mildly-elevated glycemic gap	1.98 (0.81–4.79)	0.133
Severely-elevated glycemic gap	4.58 (1.31–16.02)	0.017
**HbA1c ≥ 6.5%**		
Non-elevated glycemic gap	Reference	
Mildly-elevated glycemic gap	1.14 (0.27–4.81)	0.862
Severely-elevated glycemic gap	2.58 (0.81–8.16)	0.108

Adjusted for glycemic gap, age, sex, education, initial NIHSS, stroke subtype (TOAST), and left hemispheric lesions.

**Table 4:** Comparison of z-scores of the cognitive domains according to the glycemic gap groups.

	Non-Elevated Glycemic Gap	Mildly Elevated Glycemic Gap	Severely Elevated Glycemic Gap	* *p*-Value
	(*n* = 153)	(*n* = 102)	(*n* = 46)	
K-MMSE	−0.9 ± 2.0	−1.0 ± 2.0	−1.4 ± 1.9	0.372
Frontal	−0.9 ± 1.5	−1.1 ± 1.5	−1.9 ± 1.4	0.001
Language	−0.1 ± 1.0	−0.3 ± 1.3	−0.2 ± 1.2	0.536
Visuospatial	−1.0 ± 1.9	−1.3 ± 1.9	−1.6 ± 2.0	0.120
Memory	−1.0 ± 1.3	−0.8 ± 1.2	−1.4 ± 1.2	0.018

Abbreviations. K-MMSE: Korean Mini-Mental Status Examination. * ANCOVA was performed, adjusting for age, sex, education and initial NIHSS scores.

## Data Availability

The data are available from the corresponding author upon reasonable request.

## References

[1] Capes S.E., Hunt D., Malmberg K., Pathak P., Gerstein H.C. (2001). Stress hyperglycemia and prognosis of stroke in nondiabetic and diabetic patients: A systematic overview. Stroke.

[2] Lim J.S., Kim C., Oh M.S., Lee J.H., Jung S., Jang M.U., Lee S.H., Kim Y.J., Kim Y., Suh S.W. (2018). Effects of glycemic variability and hyperglycemia in acute ischemic stroke on post-stroke cognitive impairments. J. Diabetes Complicat..

[3] Lo J.W., Crawford J.D., Samaras K., Desmond D.W., Kohler S., Staals J., Verhey F.R.J., Bae H.J., Lee K.J., Kim B.J. (2020). Association of prediabetes and type 2 diabetes with cognitive function after stroke: A STROKOG collaboration study. Stroke.

[4] Zhang Z., Yan J., Shi H. (2013). Hyperglycemia as a risk factor of ischemic stroke. J. Drug Metab. Toxicol..

[5] Zarean E., Lattanzi S., Looha M.A., Napoli M.D., Chou S.H., Jafarli A., Torbey M., Divani A.A. (2021). Glycemic gap predicts in-hospital mortality in diabetic patients with intracerebral hemorrhage. J. Stroke Cereb. Dis..

[6] Kallani M., Mishra A., Himanshu D. (2020). Glycemic gap as a prognostic marker for critically ill patients in ICU. J. Assoc. Phys. India.

[7] Liao W.I., Wang J.C., Lin C.S., Yang C.J., Hsu C.C., Chu S.J., Chu C.M., Tsai S.H. (2019). Elevated glycemic gap predicts acute respiratory failure and in-hospital mortality in acute heart failure patients with diabetes. Sci. Rep..

[8] Liao W.I., Lin C.S., Lee C.H., Wu Y.C., Chang W.C., Hsu C.W., Wang J.C., Tsai S.H. (2016). An elevated glycemic gap is associated with adverse outcomes in diabetic patients with acute myocardial infarction. Sci. Rep..

[9] Kim Y., Lee S.H., Kim C., Kang M.K., Yoon B.W., Kim T.J., Bae J.S., Lee J.H. (2021). Personalized consideration of admission-glucose gap between estimated average and initial glucose levels on short-term stroke outcome. J. Pers. Med..

[10] Adams H.P., Bendixen B.H., Kappelle L.J., Biller J., Love B.B., Gordon D.L., Marsh E.E. (1993). Classification of subtype of acute ischemic stroke. Definitions for use in a multicenter clinical trial. TOAST. Trial of Org 10172 in Acute Stroke Treatment. Stroke.

[11] Kim B.J., Lee S.H., Jung K.H., Yu K.H., Lee B.C., Roh J.K., For Korean Stroke Registry Investigators (2012). Dynamics of obesity paradox after stroke, related to time from onset, age, and causes of death. Neurology.

[12] Nathan D.M., Kuenen J., Borg R., Zheng H., Schoenfeld D., Heine R.J., For the A1c-Derived Average Glucose (ADAG) Study Group (2008). Translating the A1C assay into estimated average glucose values. Diabetes Care.

[13] Yu K.H., Cho S.J., Oh M.S., Jung S., Lee J.H., Shin J.H., Koh I.S., Cha J.K., Park J.M., Bae H.J. (2013). Cognitive impairment evaluated with Vascular Cognitive Impairment Harmonization Standards in a multicenter prospective stroke cohort in Korea. Stroke.

[14] Hachinski V., Iadecola C., Petersen R.C., Breteler M.M., Nyenhuis D.L., Black S.E., Powers W.J., DeCarli C., Merino J.G., Kalaria R.N. (2006). National institute of neurological disorders and stroke-canadian stroke network vascular cognitive impairment harmonization standards. Stroke.

[15] Kang Y., Chin J.H., Na D.L., Lee J.H., Park J.S. (2000). A normative study of the Korean version of controlled oral word association test (COWAT) in the elderly. Korean J. Clin. Psychol..

[16] Yum T.H., Park Y.S., Oh K.J., Kim J.H., Lee Y.H. (1992). Manual for Korean-Wechsler Adult Intelligence Scale.

[17] Yi H., Chin J., Lee B.H., Kang Y., Na D.L. (2007). Development and validation of Korean version of trail making test for elderly persons. Dement. Neurocogn. Disord..

[18] Kang Y., Kim H.H., Na D.L. (1999). A short form of the Korean-boston naming test (K-BNT) for using in dementia patients. Korean J. Clin. Psychol..

[19] Kang Y., Na D.L. (2003). Professional manual; Seoul neuropsychological screening battery. Hum. Brain Res. Consult..

[20] Kang Y. (2006). A normative study of the Korean-mini mental state examination (K-MMSE) in the elderly. Korean J. Psychol..

[21] Sachdev P., Kalaria R., O’Brien J., Skoog I., Alladi S., Black S.E., Blacker D., Blazer D.G., Chen C., Chui H. (2014). Diagnostic criteria for vascular cognitive disorders: A VASCOG statement. Alzheimer Dis. Assoc. Disord..

[22] Olsen T.S. (2009). Blood glucose in acute stroke. Expert Rev. Neurother..

[23] Levine S.R., Welch K.M., Helpern J.A., Chopp M., Bruce R., Selwa J., Smith M.B. (1988). Prolonged deterioration of ischemic brain energy metabolism and acidosis associated with hyperglycemia: Human cerebral infarction studied by serial 31P NMR spectroscopy. Ann. Neurol..

[24] Siesjo B.K., Bendek G., Koide T., Westerberg E., Wieloch T. (1985). Influence of acidosis on lipid peroxidation in brain tissues in vitro. J. Cereb. Blood Flow Metab. Off. J. Int. Soc. Cereb. Blood Flow Metab..

[25] Gerstein H.C., Yusuf S. (1996). Dysglycaemia and risk of cardiovascular disease. Lancet.

[26] Vehkavaara S., Seppala-Lindroos A., Westerbacka J., Groop P.H., Yki-Jarvinen H. (1999). In vivo endothelial dysfunction characterizes patients with impaired fasting glucose. Diabetes Care.

[27] Hamilton M.G., Tranmer B.I., Auer R.N. (1995). Insulin reduction of cerebral infarction due to transient focal ischemia. J. Neurosurg..

[28] Kruyt N.D., Nys G.M., van der Worp H.B., van Zandvoort M.J., Kappelle L.J., Biessels G.J. (2008). Hyperglycemia and cognitive outcome after ischemic stroke. J. Neurol. Sci..

[29] Ergul A., Li W., Elgebaly M.M., Bruno A., Fagan S.C. (2009). Hyperglycemia, diabetes and stroke: Focus on the cerebrovasculature. Vasc. Pharm..

[30] Ryu W.S., Woo S.H., Schellingerhout D., Jang M.U., Park K.J., Hong K.S., Jeong S.W., Na J.Y., Cho K.H., Kim J.T. (2017). Stroke outcomes are worse with larger leukoaraiosis volumes. Brain.

[31] Duering M., Righart R., Csanadi E., Jouvent E., Herve D., Chabriat H., Dichgans M. (2012). Incident subcortical infarcts induce focal thinning in connected cortical regions. Neurology.

[32] de la Monte S.M., Tong M., Lester-Coll N., Plater M., Wands J.R. (2006). Therapeutic rescue of neurodegeneration in experimental type 3 diabetes: Relevance to Alzheimer’s disease. J. Alzheimers Dis..

[33] Takechi R., Lam V., Brook E., Giles C., Fimognari N., Mooranian A., Al-Salami H., Coulson S.H., Nesbit M., Mamo J.C.L. (2017). Blood-brain barrier dysfunction precedes cognitive decline and neurodegeneration in diabetic insulin resistant mouse model: An implication for causal link. Front. Aging Neurosci..

[34] Kellar D., Lockhart S.N., Aisen P., Raman R., Rissman R.A., Brewer J., Craft S. (2021). Intranasal Insulin reduces white matter hyperintensity progression in association with improvements in cognition and CSF biomarker profiles in mild cognitive impairment and Alzheimer’s disease. J. Prev. Alzheimers Dis..

[35] den Heijer T., Vermeer S.E., van Dijk E.J., Prins N.D., Koudstaal P.J., Hofman A., Breteler M.M. (2003). Type 2 diabetes and atrophy of medial temporal lobe structures on brain MRI. Diabetologia.

